# Magnetic Driven Nanocarriers for pH-Responsive Doxorubicin Release in Cancer Therapy

**DOI:** 10.3390/molecules25020333

**Published:** 2020-01-14

**Authors:** João Nogueira, Sofia F. Soares, Carlos O. Amorim, João S. Amaral, Cláudia Silva, Fátima Martel, Tito Trindade, Ana L. Daniel-da-Silva

**Affiliations:** 1 CICECO-Aveiro Institute of Materials, Department of Chemistry, University of Aveiro, 3810-193 Aveiro, Portugal; jh.nogueira@ua.pt (J.N.); sofiafsoares@ua.pt (S.F.S.); tito@ua.pt (T.T.); 2 CICECO-Aveiro Institute of Materials, Department of Physics, University of Aveiro, 3810-193 Aveiro, Portugal; amorim5@ua.pt (C.O.A.); jamaral@ua.pt (J.S.A.); 3 Unit of Biochemistry, Department of Biomedicine, Faculty of Medicine, University of Porto, 4200-319 Porto, Portugal; rakelclaudia@hotmail.com (C.S.); fmartel@med.up.pt (F.M.); 4 i3S-Instituto de Investigação e Inovação em Saúde, Universidade do Porto, 4200-135 Porto, Portugal

**Keywords:** doxorubicin, magnetic nanoparticles, iron oxide, κ-carrageenan, drug carrier, pH-responsive

## Abstract

Doxorubicin is one of the most widely used anti-cancer drugs, but side effects and selectivity problems create a demand for alternative drug delivery systems. Herein we describe a hybrid magnetic nanomaterial as a pH-dependent doxorubicin release carrier. This nanocarrier comprises magnetic iron oxide cores with a diameter of 10 nm, enveloped in a hybrid material made of siliceous shells and ĸ-carrageenan. The hybrid shells possess high drug loading capacity and a favorable drug release profile, while the iron oxide cores allows easy manipulation via an external magnetic field. The pH responsiveness was assessed in phosphate buffers at pH levels equivalent to those of blood (pH 7.4) and tumor microenvironment (pH 4.2 and 5). The nanoparticles have a loading capacity of up to 12.3 wt.% and a release profile of 80% in 5 h at acidic pH versus 25% at blood pH. In vitro drug delivery tests on human breast cancer and non-cancer cellular cultures have shown that, compared to the free drug, the loaded nanocarriers have comparable antiproliferative effect but a less intense cytotoxic effect, especially in the non-cancer cell line. The results show a clear potential for these new hybrid nanomaterials as alternative drug carriers for doxorubicin.

## 1. Introduction

**Scheme 1:**
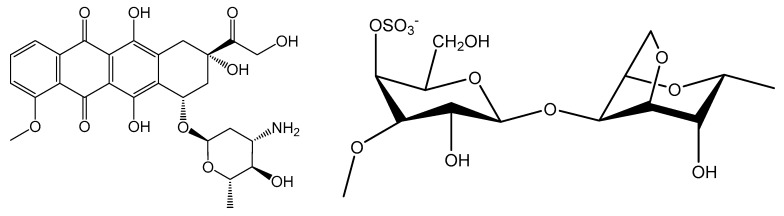
Chemical structure of doxorubicin (**left**) and disaccharide unit of κ-carrageenan (**right**).

Doxorubicin (DOX, Scheme 1) is an anthracycline antitumor agent, one of the most common and effective anticancer drugs used in the treatment of various forms of cancer such as lymphomas, sarcomas and breast cancers [1]. The latter in particular is the most common cancer in women, and doxorubicin is one of the main treatments for early and advanced breast cancers [2]. However, doxorubicin presents a number of drawbacks. About 40% of the drug and its metabolites are excreted, and thus, only a small amount of drug actually reaches and acts on the tumor target site [3]. It also causes a notable, well documented cardiac toxicity [4,5,6]. Another major complication is the possible development of drug resistance mechanisms. Tumor drug resistance contributes to a 90% failure rate of chemotherapy treatments of metastatic disease [7]. Drug resistance can occur via over-expression of cell surface pumps (such as the ATP-binding cassette superfamily) [8], or via changes in DNA repair and cellular apoptosis mechanisms [9].

To overcome these chemotherapy issues, one of the promising avenues is the use of nanostructured platforms as drug delivery systems [10,11,12]. Nanosized drug delivery systems can carry drugs to tumor cells, then penetrate physiological barriers, thus protecting the drug from biological clearance, bypass drug resistance mechanisms and finally release the drug specifically on tumor, namely after triggering by a specific stimulus [3,13]. A drug-loaded carrier can accumulate on tumor sites by exploiting the enhanced permeability and retention (EPR) effect, first described in 1986 [14], where tumor vascular permeability is greatly increased compared with healthy tissue [15]. This effect occurs in solid tumors leading to enhanced diffusion of macromolecules and nanoparticles into the tumor. Owing to this effect, it is possible to explore passive tumor targeting by drug-loaded nanoparticles, having a particle size range between 10 and 100 nm, which has been reported to be optimal for in vivo targeting delivery [13,16]. Additional features can be added to the carrier aiming to enhance drug delivery performance. For example, the use of pH-responsive biopolymers such as chitosan led to carriers that provide enhanced drug release in the tumor acidic microenvironment (due to anaerobic glucose metabolism) and in acidic intracellular organelles (such as endosomes and lysosomes), over release in blood [3,17,18,19,20,21]. Magnetic nanocarriers, either individual or as components of hybrid nanomaterials [16,22,23,24,25,26], have also been recognized as drug release vectors having with several advantages [10,27]. Indeed, magnetic nanocarriers can be guided towards target tissues under a magnetic gradient and eventually be used as contrast agents for magnetic resonance imaging (MRI), thus allowing for simultaneous cancer treatment and monitoring [24,26]. Furthermore, internalized magnetic nanocarriers, when exposed to an alternative magnetic field, increase locally the temperature, which allows us to exploit in a single platform a therapeutic action (magnetic hyperthermia) combined with drug delivery [28,29].

In the context of doxorubicin based therapies, and in particular its use in breast cancer treatment, several studies have reported the use of nanocarriers to enhance doxorubicin-based treatment [2]. Hence, liposomal doxorubicin, a system where DOX is encapsulated in a spherical lipid-based vesicle, has several variants, either in the market or in clinical stages [30]. Doxil^®^ is a polyethylene glycol (PEG) based form of liposomal DOX. Known also as Caelyx^®^, was the first market-approved nanoformulation containing doxorubicin. As compared to free DOX, it shows comparable efficiency against metastatic breast cancer, but presents a lower risk of cardiac damage. However, Doxil^®^ presents other side effects, notably palmar-plantar erythrodysteria (PPE), also known as foot-and-hand syndrome, characterized by redness, tenderness and peeling of the skin [31]. Another possible side effect is an often mild, but possibly severe, allergic reaction caused by some of the formulation’s compounds [32]. A commercialized, non-PEGylated but still liposomal alternative is Myocet^®^, using a phosphatidylcholine and cholesterol membrane. While having a lower incidence of PPE, the overall survival rate was not improved, and the formulation has worse hematological toxicity and a higher risk of neutropenia [33]. The final liposomal DOX formulation in the market is Lipo-Dox^®^, which includes PEGylated liposomes as well; however, it fails to show improvements over Doxil^®^ regarding efficacy and toxicity [34].

Owing to the drawbacks above mentioned, there is a demand for more effective nanocarriers for doxorubicin. In the context of breast cancer treatment some attempts have been reported using nanoscale systems, including chitosan nanoparticles [35], organosilicon nanoparticles [36,37] and magnetic iron oxide nanocarriers [38]. Magnetic carriers comprising iron oxide nanoparticles functionalized with organic coatings, including azo [23], chitosan [17], polymethacrylic acid [39] and carboxymethyl cellulose [40] are also being investigated. To the best of our knowledge, magnetic nanocarriers based on ĸ-Carrageenan shells have not been explored for DOX release in cancer therapies.

Carrageenans are a family of linear sulfated polysaccharides (100–1000 kDa) extracted from marine red algae (Rhodophyceae), widely used as an emulsifier, stabilizers and thickeners in the food and pharmaceutical industries. In human nutrition, they are a good source of dietary fiber and can reduce serum cholesterol and triglyceride levels [41]. Carrageenans have very low toxicity in humans but are known for an inflammatory effect in some rodents, being frequently used for inflammatory induction in toxicology studies in vivo and have also shown anticoagulant, antiviral and antitumor activities [42]. ĸ-Carrageenan (Scheme 1), composed of d-galactose and 3,6-anhydrogalactose units that bears one sulfate group per disaccharide unit [43,44]. Carrageenan-based micro- and nano-scale delivery systems have been reported, often taking advantage of the sulfate content, which results in a negative surface charge that promotes an uptake of cationic target molecules or facilitates functionalization with positively charged components for complex drug carriers [43,45]. Examples of systems reported in the literature include pH-responsive hydrogels for intestinal drug delivery [46], hybrid calcium-biopolymer microparticles for DOX delivery [47,48] and ĸ-carrageenan-coated γ-Fe_2_O_3_ nanoparticles with chemotherapeutic potential [49]. While there has been research on the combination of magnetic particles with carrageenan for drug delivery [27], there are no reported works combining such systems with DOX.

In the past few years, our research group has developed a new strategy for the preparation of core/shell spheroidal nanoparticles, comprising magnetite cores (Fe_3_O_4_) coated with amorphous silica shells [50], or with siliceous shells enriched in polymers from renewable sources including starch [51], alginate [52], chitosan [53,54] and carrageenan [51,55]. We aimed at creating magnetic bionanocomposites whose ability to interact with target molecular species depends on the chemical functionalities of the shells. In this work, κ-carrageenan based magnetic hybrid nanoparticles were investigated in the controlled release of DOX. The hybrid particles were tested for pH-responsive DOX release and for the cytotoxic activity against human breast cancer and non-cancer cell lines. The loaded hybrid particles have successfully shown to favor drug release at acidic conditions, as those found in tumor microenvironments, and to cause cellular damage to human cell lines.

## 2. Results and Discussion

### 2.1. Characterization of the Magnetic Nanocarriers

**Figure 1:**
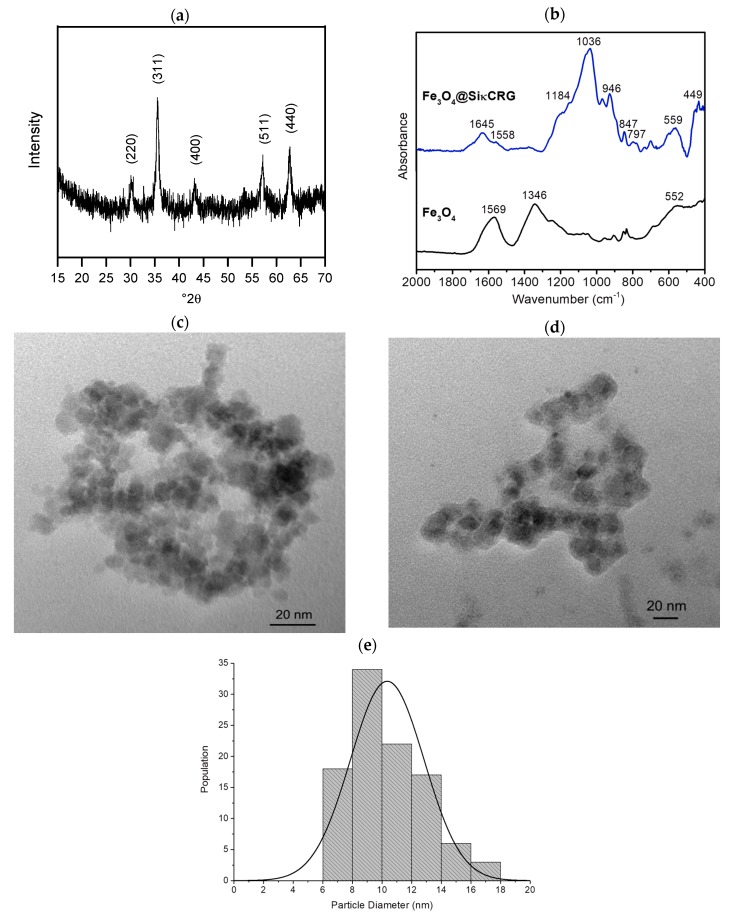
Physicochemical characterization of nanoparticles. (**a**) XRD diffraction pattern, with Bragg reflections identified. (**b**) ATR-FTIR spectra, with main bands identified. TEM images of Fe_3_O_4_ (**c**) and Fe_3_O_4_@SiκCRG (**d**) nanoparticles. (**e**) Histogram for size distribution of Fe_3_O_4_ core nanoparticles.

The powder XRD pattern of the magnetic cores (Figure 1a) matched the diffraction patterns reported for cubic magnetite (Fe_3_O_4_) [56]. The FTIR spectrum of Fe_3_O_4_ (Figure 1b) shows a strong band at 552 cm^−1^ characteristic of the stretching vibration of the magnetite lattice [57]. This vibrational mode is also observed as a band slightly shifted 559 cm^−1^, in the FTIR spectrum of the coated Fe_3_O_4_ particles. Two other vibrational are observed in the FTIR spectrum of Fe_3_O_4_, one due to the stretching of –CO groups at 1349 cm^−1^ and the other one due to the asymmetric stretching of –COO^−^ groups at 1569 cm^−1^, both owed to the surface citrate ions that stabilize the colloidal magnetic nanoparticles [50]. The FTIR spectrum of Fe_3_O_4_@SiĸCRG particles displays the characteristic vibrational bands of ĸ-carrageenan in the region 1000–1100 cm^−1^ (C–O and C–OH stretching vibrations), at 847 cm^−1^ (α(1-3)-d-galactose C–O–S stretching vibration) and at 1184 cm^−1^ (O=S=O asymmetric stretching of sulfonate groups) [58]. The strong band at 449 cm^−1^, is ascribed to the O–Si–O deformation [59], which is in agreement with the formation of a siliceous network coating the iron oxide cores. Despite overlapping with vibrational bands from the biopolymer, the bands assigned to amorphous silica [51,60] are observed at 797 and 1036 cm^−1^ due respectively to symmetric and asymmetric Si–O–Si stretching and at 946 cm^−1^ due to Si–OH stretching. The band at 1558 cm^−1^ is ascribed to N–H bending modes coupled to C–N stretching, which confirms the covalent bonding between the carrageenan and the siliceous network through a urethane linkage [61,62]. The band at 1645 cm^−1^ can be either ascribed to adsorbed water (H_2_O bending mode) or due to the stretching vibration of hydrogen-bonded C=O in urea, [63] thus indicating the coexistence of urea and urethane groups in the hybrid shells [50,51,55,60].

Figure 1c,d show the morphological characteristics of the nanoparticles as assessed by transmission electron microscopy (TEM). The TEM image of the Fe_3_O_4_ particles shows spheroidal nanoparticles with an average size of 10 ± 2.5 nm, as shown in the particle size histogram (Figure 1e). The TEM images of the coated particles show a thin (5.2 ± 2.1 nm) outer-layer corresponding to the siliceous shell encapsulating the magnetic cores. Elemental microanalysis of the Fe_3_O_4_@SiĸCRG particles (Table 1) indicates the presence of sulphur, which originates from the sulfate groups of the biopolymer that forms the coating. Furthermore, the content of carbon, which in the bare Fe_3_O_4_ is due to citrate employed for colloidal stabilization, markedly increased after coating the particles. These results are in agreement with the presence of the sulfated polysaccharide ĸ-carrageenan in the coating shells. Note that functionalized used here yielded a marked higher sulphur content (1.27 wt.%) when compared with the results obtained via the conventional multi-step functionalization by covalent grafting of ĸ-carrageenan to amine functionalized silica coated Fe_3_O_4_ nanoparticles (0.08 wt.%) [64]. These results are consistent with higher biopolymer content at the surface of the nanoparticles using a surface modification approach based on the hydrolytic condensation of the ĸ-carrageenan Si-alkoxide derivative.

The BET specific surface area was determined by N_2_ adsorption data in the relative pressure (p/p^0^) range 0.01–0.20, and decreased from 151.95 to 13.94 m^2^ g^−1^ after coating (Table 1). This decrease can be explained by the increase in overall particle size after biopolymer incorporation. [51]

**Figure 2:**
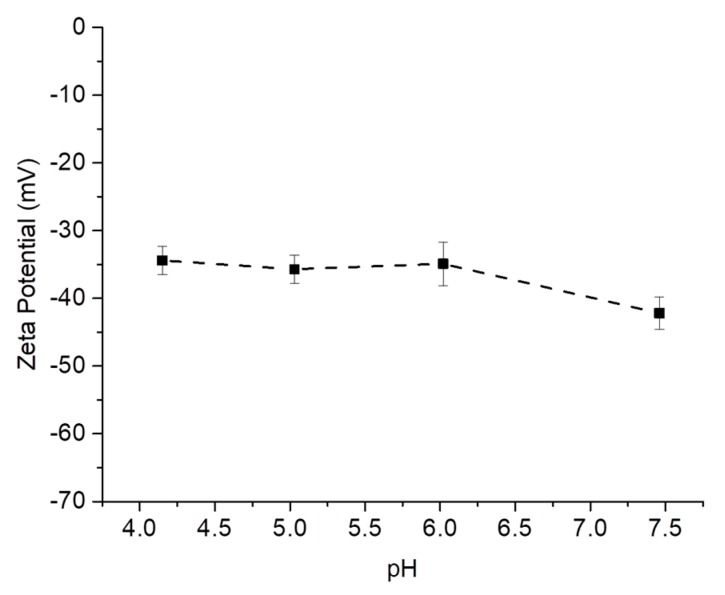
Zeta potential titration for Fe_3_O_4_@SiκCRG particles.

The surface charge of the Fe_3_O_4_@SiĸCRG particles was assessed by zeta potential measurements at variable pH. The zeta potential was markedly negative, ranging from −34.4 ± 2.1 at pH = 4.2 to −42.2 ± 2.4 mV at pH = 7.4 (Figure 2), which is consistent with the presence of sulfonate groups from carrageenan at the surface of the coated magnetic particles. It is worth noting that in this case the negatively charged surface are highly desirable because they will favor electrostatic interactions with the protonated primary amine groups of doxorubicin, at acidic pH, this promoting the loading of DOX. Furthermore, it should be stressed that at pH 7.4 (physiological medium) the colloidal particles still show a negative zeta potential value that imparts colloidal stability due to interparticle electrostatic repulsions.

**Figure 3:**
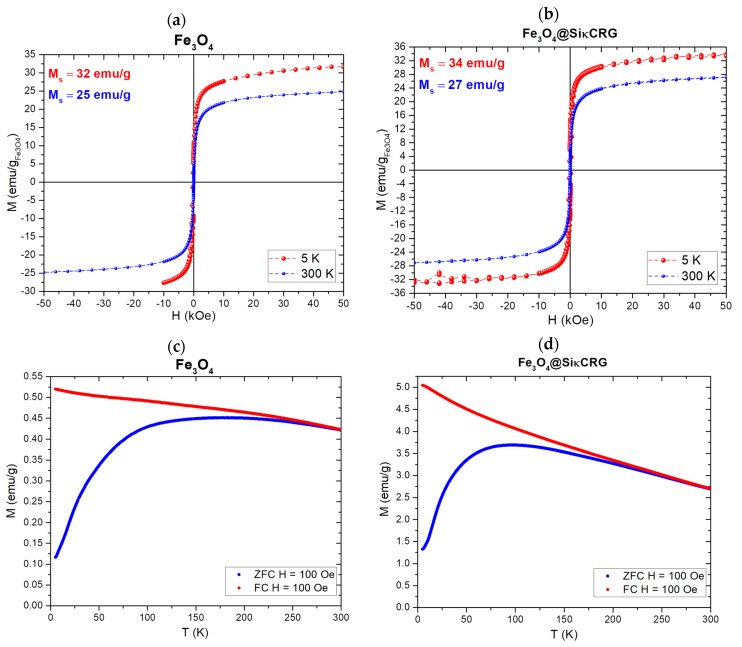
Field dependent magnetization curves (normalized for iron oxide content) of Fe_3_O_4_ nanoparticles (**a**) and Fe_3_O_4_@SiκCRG nanoparticles (**b**). Temperature dependent magnetization curves of Fe_3_O_4_ nanoparticles (**c**) and Fe_3_O_4_@SiĸCRG nanoparticles (**d**).

Magnetic measurements were performed in a Quantum Design MPMS3 magnetometer for both magnetite and the corresponding coated particles. The field dependence of the magnetization normalized with the iron oxide content of the nanoparticles measured at 300 K and 5 K is displayed in Figure 3a,b (non-normalized curves are shown in Figure S2, Supporting Information). The parameters coercivity (*H_c_*), remanent magnetization (*M_r_*) and saturation magnetization (*M_s_*) values obtained from the magnetization curves at 300 K are listed in Table 2. Both bare and coated particles exhibited superparamagnetic-like behavior at room temperature with a hysteresis loop, residual remnant magnetization and coercive field values compatible with the effect of a remnant field of the superconducting coils. This behavior is expected due to the small particle size of the nanoparticles. The observed magnetization saturation was of 25 and 27 emu/g Fe_3_O_4_ at 300 K; 32 and 34 emu/g Fe_3_O_4_ at 5 K, for bare and coated nanoparticles, respectively. Though magnetic properties of magnetite nanoparticles are dependent on particle size, these values are lower than those typically found in high purity magnetite nanoparticles of similar sizes [65]. These differences may be explained by incomplete crystallization of magnetite, or irregularities in the spheroidal shapes of the particles [66]. The Fe_3_O_4_@SiĸCRG particles show an almost identical magnetic saturation when compared to bare nanoparticles. Magnetization versus applied magnetic field data at 5 K show coercivity values around 250 Oe. This suggests a blocking temperature above this temperature, as confirmed via zero-field cooled and field-cooled measurements, shown in Figure 3c,d. The lower blocking temperatures of the Fe_3_O_4_@SiĸCRG particles hint at the effect of the coating on the kinetics of the particles during magnetic relaxation. Due to their superparamagnetic quality, the small particles have not only the potential to be a drug release system but also to be used for magnetic resonance imaging (MRI), which translates to a possible theranostic application. It is thus of high interest that these particles show an adequate drug loading and release profile.

### 2.2. Doxorubicin Loading

**Figure 4:**
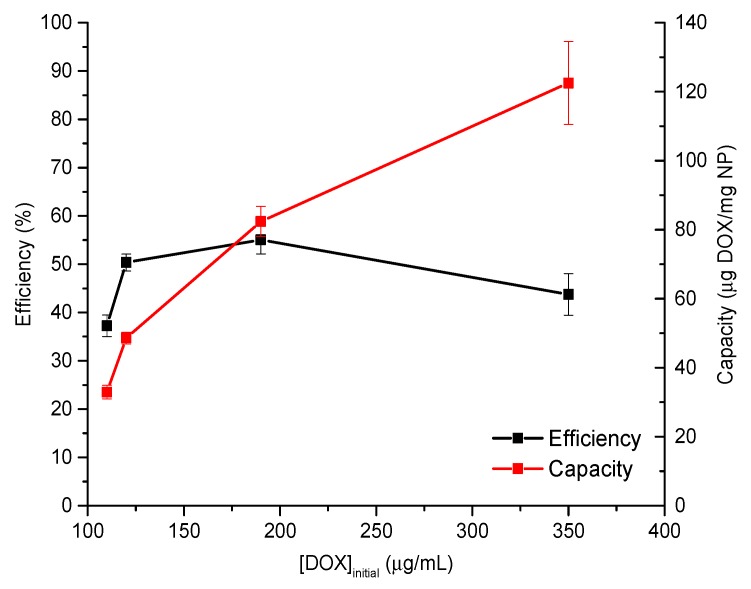
Uptake curve for doxorubicin, expressed in loading efficiency (%) and nanoparticle capacity (μg DOX/mg NP). pH = 6, [NP] = 1.25 mg/mL.

The nanoparticles Fe_3_O_4_@SiĸCRG were loaded with DOX using a buffer solution at pH = 6, DOX concentration ranging from 110 to 350 μg/mL. The results of loading efficiency and capacity are shown in Table S1. The loading efficiency ranged from 37% to 55% and the loading capacity from 33 to 123 μg/mg (3.3–12.3 wt.%) and has increased with initial DOX concentration in the aqueous solution (Figure 4). These results were similar to values obtained in previous studies using other magnetic carriers, such as chitosan-coated magnetite nanoparticles (18 wt.%) [17] and magnetic polymersomes (12 wt.%) [67].

**Figure 5:**
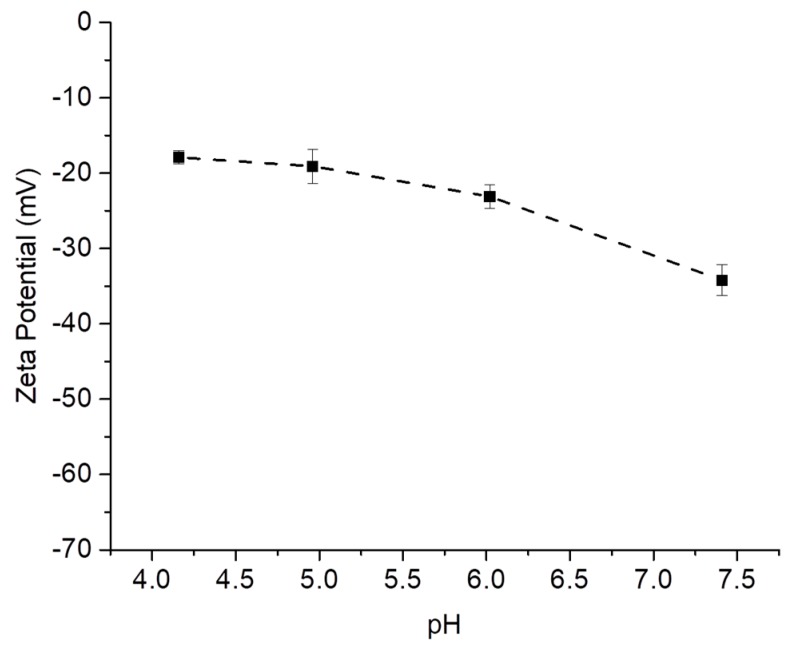
Zeta potential titration for Fe_3_O_4_@SiκCRG particles loaded with doxorubicin (loading capacity was 3.2 wt.%).

In aqueous solution, DOX molecules adopt different conformations depending on the solution pH (Figure S3, Supporting Information) [68]. At pH 6, it is expected that drug loading is promoted by electrostatic interactions between the primary amine groups of DOX and the sulfonate groups of the carrageenan. Moreover, hydrogen-bonding interactions between OH groups of carrageenan/siliceous network and hydroxyl groups of the anthraquinone ring in DOX will also favor drug loading at this pH. Previous studies have indicated this type of interactions is of uttermost relevance in the formation of complexes between DOX and dextran sulfate, which is also a sulfated polysaccharide [69]. There are also π–π stacking interactions between the aromatic groups of DOX molecules being brought close together, which also promotes drug loading [69,70]. As expected, with DOX loading the surface of the nanoparticles becomes less negative. Still, the zeta potential of loaded particles ranged from −17.9 ± 0.9 mV at pH = 4.2 to −34.2 ± 2.0 mV at pH = 7.4 (Figure 5), which is indicative of moderate colloidal stability [71]. It is worth noting that the zeta potential was assessed in buffers. In biological environment and in cell cultures medium the adsorption of proteins may alter the surface properties of the nanoparticles and influence the colloidal stability.

### 2.3. Doxorubicin pH Mediated Release Studies

**Figure 6:**
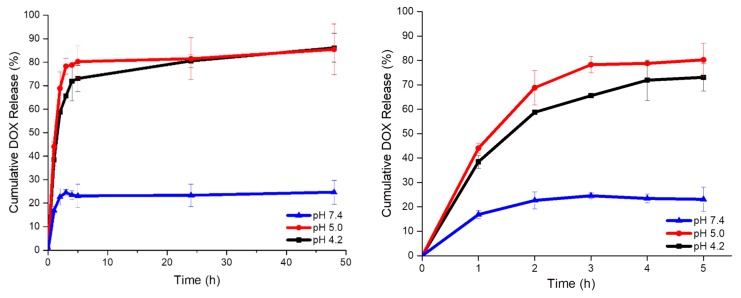
Doxorubicin release over time from loaded Fe_3_O_4_@ĸCRG carriers, at 37 °C and variable pH (loading capacity was 12.3 wt.%). Full profile for 48 h (**left**) and first 5 h (**right**).

Figure 6 shows the cumulative release of DOX from the Fe_3_O_4_@Si CRG nanocarriers at pH 4.2, 5.0 and 7.4, in buffered solutions. After 1 h, a burst release occurs in all formulations and the equilibrium is reached after 5 h, at pH 7.4 and pH 5. At pH 4.2 the release of DOX was gradually prolonged up to 48 h. The DOX release was pH-dependent being markedly faster in acidic conditions. Thus, after 5 h the DOX release was approximately 73% and 80% in acidic conditions (pH 4.2 and 5, respectively) and 25% at pH 7.4. Similar behavior has been observed for pH-responsive DOX nanocarriers based on chitosan coated magnetic nanoparticles [17]. This pH-sensitive behavior of Fe_3_O_4_@SiĸCRG nanocarriers is of high interest for the controlled release of may benefit the release of doxorubicin in tumor cells because the intracellular pH is lower than in the healthy cells [72].

**Figure 7:**
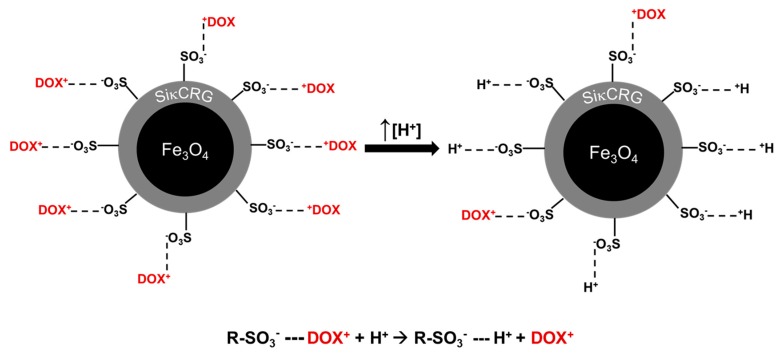
Schematic representation of the proposed cation exchange mechanism behind DOX release from the nanocarriers in acidic conditions, with the equation showing the equilibrium shift.

It is likely that the release of DOX occurs through a cation exchange mechanism (Figure 7) because surface charge of the nanocarriers is negative, not varying significantly across the work pH range (Figure 2), and DOX is cationic in acidic conditions. The acidic environment promotes the exchange of protons between the primary amine groups of DOX and the sulfonate groups of κ-carrageenan [73]. The DOX release was slightly pronounced at pH 5 than at pH 4.2 for the first five hours but became equalized within a 24 h period. We interpreted the faster initial release at pH 5 as due to a polymer swelling effect. The outer shell of the nanocarriers contain ĸ-carrageenan in its composition, which is well known to form hydrogels with a pH-dependent swelling behavior [74]. Although in these nanocarriers the ĸ-carrageenan was grafted to the siliceous network forming the outer shells, there were still domains enriched in ĸ-carrageenan that can undergo swelling in an aqueous medium. Carrageenan hydrogels experience increased swelling when the pH increased from acidic to neutral conditions. This swelling effect can enhance the drug release by favoring the diffusion of DOX molecules within the hybrid network [74,75].

It is the strong swelling behavior experienced by carrageenan at neutral pH that also contributes to DOX release at pH 7.4. In this case, the release of DOX was slower due to a less extent of protonation, which results in lower competition of the protons for the sulphonated sites of ĸ-carrageenan. Nevertheless, there is still a 25% drug release because at this pH there will be a number of neutral DOX molecules less prone to interact with sulfonate groups at the surface of the magnetic nanocarriers.

The release kinetic data were reasonably described by the Weibull model (equation S1) with a coefficient of determination (R^2^) in the range 0.926–0.958 (Supporting Information, Figure S4 and Table S2). This is a general empirical equation that has been successfully applied to several drug release systems [76,77].

### 2.4. Antiproliferative and Cytotoxicity In Vitro Evaluation

**Figure 8:**
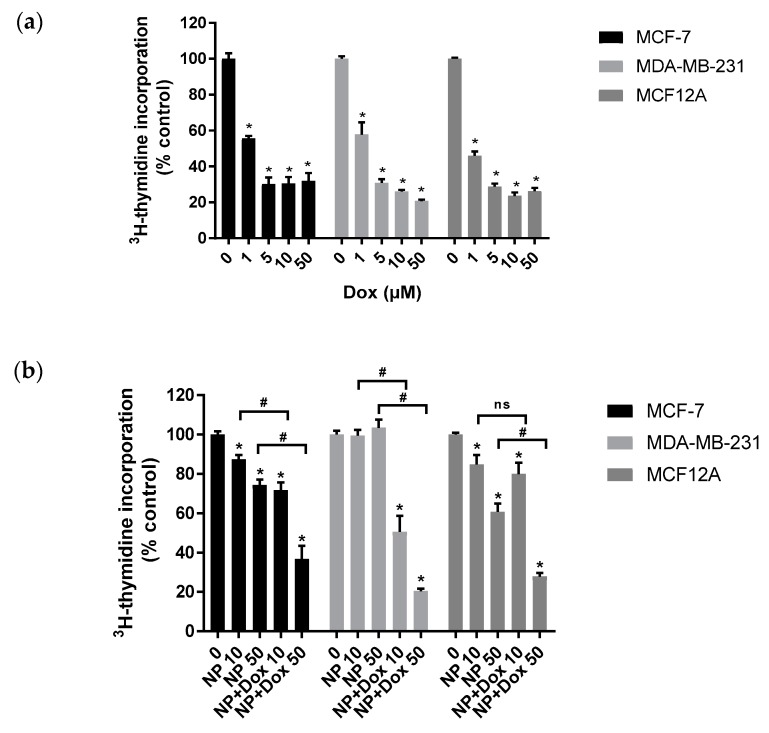
Cell proliferation after exposure for 24 h to (**a**) free doxorubicin (μM) and (**b**) unloaded nanoparticles (NP) and nanoparticles loaded with DOX (NP + DOX; μM), for all cell lines. Shown are arithmetic means ± SEM (*n* = 6). (**a**) * significantly different from control (*p* < 0.05; Student’s *t* test). (**b**) * significantly different from control; ^#^ significantly different from each other (*p* < 0.05; ANOVA + Student-Neuman–Keuls test); ^ns^ not significant (*p* < 0.05; ANOVA + Student-Neuman–Keuls test).

**Figure 9:**
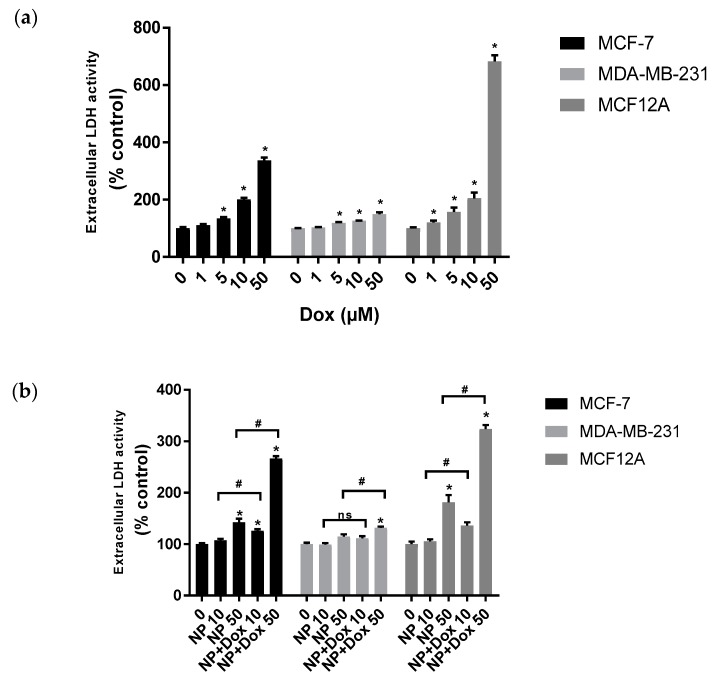
Cell viability after exposure for 24 h to (**a**) free doxorubicin (μM) and to (**b**) unloaded nanoparticles (NP) and nanoparticles loaded with DOX (NP + DOX; μM), for all cellular lines. Shown are arithmetic means ± SEM (*n* = 8). (**a**) * significantly different from control (*p* < 0.05) (Student’s *t* test). (**b**) * significantly different from control; ^#^ significantly different from each other (*p* < 0.05; ANOVA + Student-Neuman–Keuls test); ^ns^ not significant (*p* < 0.05; ANOVA + Student-Neuman–Keuls test).

The proliferation and viability of the cells after their exposure to free doxorubicin (DOX), unloaded nanoparticles (NP) and nanoparticles loaded with doxorubicin (NP + DOX) are shown in Figure 8 and Figure 9, respectively. The tests were performed in MCF-7 and MDA-MB-231 human breast cancer cells and in MCF12A non-cancerous human breast epithelial cells.

As expected, free DOX has a concentration-dependent antiproliferative effect on all cell lines (Figure 8a). Loaded nanoparticles also present a concentration-dependent antiproliferative effect on all cell lines, and this effect was more marked than the effect of unloaded nanoparticles (Figure 8b). The antiproliferative effect of DOX-loaded NPs is similar to that of free DOX at DOX concentrations of 50 μM, in all cell lines (Figure 8).

As expected, free DOX shows a concentration-dependent cytotoxic effect on all cell lines (Figure 9a). Both cancer cell lines (MCF-7 and MDA-MB-231) and the non-cancerous cell line (MCF12A) suffer deleterious cytotoxic effects when exposed to loaded nanoparticles, and again, this effect is much more marked than the effect observed with unloaded nanoparticles (Figure 9b). The cytotoxic effect of DOX-NPs is lower than that of DOX itself in all the three cell lines, but this is especially evident in the non-cancerous cell line (Figure 9).

When comparing the effect of NP + DOX in the different cell lines, we observed that its antiproliferative effect was equally potent in the three cell lines (Figure 8b), but its cytotoxic effect was less potent in MDA-MB-231 cells than in the other cell lines (Figure 9b). So, the antiproliferative and cytotoxic effect of NP-DOX do not appear to be cell type-specific. This is not surprising as the NP + DOX effect was not meant to be selective according to cell type since all cells possess acidic endosomes that can provide conditions for DOX release once the particles are taken up, but rather its selectivity is due to local tumor vascularity and acidic tumor microenvironment when in physiological conditions. The loaded nanoparticles are expected to leave the bloodstream and enter in contact with cancer cells via the EPR effect and then begin releasing DOX in their vicinity due to a local decrease in pH.

When comparing the effect of DOX in the three cell lines used, we concluded that the non-cancerous cell line (MCF12A) appeared to be more susceptible to DOX-induced cytotoxicity, but not more susceptible to the antiproliferative effect than the cancer cell lines (MCF-7 and MDA-MB-231; Figure 8 and Figure 9). Moreover, of the cancer cell lines, MDA-MB-231 appears more resistant to the cytotoxic effect of DOX than MCF-7 cells, but both cell lines are equally vulnerable to its antiproliferative effect. In other words, susceptibility to the cytotoxic effect of DOX varies in the distinct cell lines, but not vulnerability to its antiproliferative effect. This is in line with doxorubicin’s mechanism of action: it disrupts topoisomerase, preventing cellular replication and ultimately inducing programmed cellular death. The specific cytotoxic damage and resulting form of cell death induced by doxorubicin varies with concentration, treatment duration, specific form of cancer and drug resistance [78]. Both MDA-MB-231 [79] and MCF-7 [78] cell lines have shown the ability to gain resistance to doxorubicin. Nevertheless, the opposite can happen and some studies have shown a lower IC_50_ for DOX in MDA-MB-231 than in MCF-7 cells [80,81]. Different rates of nanoparticles uptake, depending on cell type, may also be at play to explain this. Nonetheless, it is clearly shown that DOX-NPs disrupts cellular division and reduces cell viability to a comparable level to free doxorubicin, and that, differently from what is found with DOX, its antiproliferative effect is not more marked in non-cancerous cells. Our results thus show the potential of the hybrid nanoparticles as a novel anticancer drug delivery system.

## 3. Materials and Methods

### 3.1. Materials

Doxorubicin hydrochloride (≥95%; DOX HCl) was purchased from Thermo Fisher (Waltham, MA, USA). κ-Carrageenan (κCRG), 3-(triethoxysilyl)propyl isocyanate (ICPTES) (95%), ethanol (CH_3_CH_2_OH; absolute) and acetone (C_3_H_6_O; 100%) were purchased from Honeywell Fluka (Seelze, Germany). Dimethylformamide (DMF) [HCON(CH_3_)_2_] was purchased from Carlo Erba Reagents (Chaussée du Vexin, France). Tetraethyl orthosilicate (TEOS; 99%), iron (III) chloride hexahydrate (FeCl_3_·6H_2_O; 99%), iron (II) chloride tetrahydrate (FeCl_2_·4H_2_O; 99%), sodium citrate tribasic dihydrate (Na_3_C_6_H_5_O_7_·2H_2_O; 99%), nitric acid (HNO_3_; 65%), sodium phosphate dibasic anhydrous (Na_2_HPO_4_; 99%) and sodium phosphate monobasic (NaH_2_PO_4_; 99%) were purchased from Sigma-Aldrich (St. Louis, MO, USA). Sodium hydroxide (NaOH; 99%) and potassium hydroxide (KOH; >86%) were purchased from Labchem (Zelienople, PA, USA). Ammonia (NH_4_; 25%) and methanol (CH_3_OH; solvent) were purchased from VWR (Radnor, PA, USA). Milli-Q water was obtained from the Synergy equipment from Millipore (Burlington, MA, USA) with a 0.22 µm filter.

^3^H-thymidine ([methyl-3*H*]thymidine; specific activity 79.0 Ci/mmol) was purchased from GE Healthcare GmbH (Freiburg, Germany). DMEM:Ham’s F12 medium (1:1; catalogue #FG4815) was purchased from Biochrom (Berlin, Germany). Human epidermal growth factor, MEM (catalogue #31095-052) was purchased from Gibco (Thermo Fisher, Waltham, MA, USA). Cholera toxin (95%), heat-inactivated horse serum, bovine insulin, hydrocortisone (98%), nicotinamide adenine dinucleotide (NADH; ≥95%), sodium pyruvate (99%), trichloroacetic acid (TCA; 99%), antibiotic/antimycotic solution (100 U mL^−1^ penicillin, 100 μg mL^−1^ streptomycin and 0.25 μg mL^−1^, amphotericin B), FBS (fetal bovine serum), l-glutamine, HEPES (*N*-2-hydroxyethylpiperazine-*N*′-2-ethanesulfonic acid) (99.5%), methylthiazolyldiphenyl-tetrazolium bromide (MTT; 98%), RPMI 1640 (catalogue #R6504), trypsin–EDTA solution (0.25%), Triton X-100 and PBS (phosphate buffered saline) were purchased from Sigma (St. Louis, MO, USA). The compounds to be tested were dissolved in water; controls were run in the presence of the respective solvent.

### 3.2. Synthesis of the Magnetic Nanocarriers

**Scheme 2:**
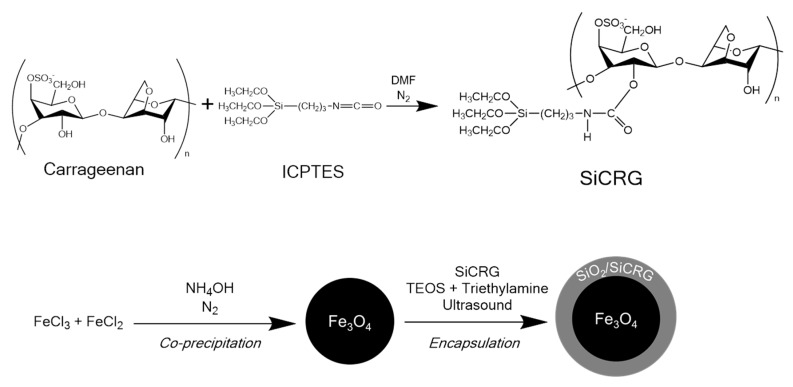
Preparation of siliceous carrageenan coated iron oxide nanoparticles.

The synthesis of magnetic carriers (Fe_3_O_4_@SiĸCRG) comprises two distinct steps. The first stage consists of the synthesis of the magnetic core (magnetite—Fe_3_O_4_) by the co-precipitation method. Then Fe_3_O_4_ particles were encapsulated in shells of a siliceous hybrid material containing κ-carrageenan covalently linked to the network, with an adaptation of the Stöber method. The procedure is summarized in Scheme 2.

Spherical superparamagnetic magnetite nanoparticles with an average size of 10 nm were synthesized by co-precipitation of ferric (Fe^3+^) and ferrous (Fe^2+^) ions and then stabilized with citrate ions [50]. In a typical procedure, 4.43 g of FeCl_3_.6H_2_O and 1.625 g of FeCl_2_.4H_2_O was dissolved in 190 mL of deoxygenated ultrapure water under a N_2_ atmosphere with mechanical stirring (650 rpm). To start the reaction, 10 mL of ammonia was added and stirred for 10 min. The nanoparticles were magnetically separated and washed with distilled water, then washed twice with HNO_3_ (2 M), with magnetic separation. The particles were dispersed in 200 mL water, and the pH was adjusted to 2.5 with a few drops of NaOH (1 M). Sodium citrate (5 mL, 0.5 M) was added to the suspension, which was mechanically stirred for 1 h. The nanoparticles were magnetically recovered and then freeze-dried.

For the encapsulation, 100 mg of Fe_3_O_4_ particles were dispersed in 18 mL of ultra-pure water, with sonication for 10 min. The biopolymer ĸ-carrageenan was modified with alkoxysilyl groups by reaction with ICPTES [55]. A mixture comprising the silicon derivative of carrageenan (0.4 g), TEOS (0.4 mL) and ethanol (3 mL) was added to the dispersion. Triethylamine (0.1 mL) was added as catalysts. The mixture was placed in an ice bath and was sonicated (Vibracell, Sonics & Materials, Newtown, CT, USA) for 15 min. The resulting particles were collected magnetically, washed with acetone and ethanol and dried at room temperature.

### 3.3. Nanoparticle Characterization

The textural properties of the hybrids were assessed by nitrogen physisorption performed with a Gemini V2.0 Micromeritics Instruments (Micromeritics, Norcross, GA, USA). The specific surface area of the particles was assessed by N_2_ adsorption isotherm measurements performed with a Gemini V2.0 Micromeritics instruments at −196 °C. The specific surface area was determined using the Brunauer–Emmett–Teller (BET) equation for relative pressures (p/p_0_) up to 0.3 [82]. Prior to BET measurements, the samples were degassed at 80 °C under nitrogen flow overnight. Elemental analysis of carbon, sulphur and hydrogen was obtained on a Leco Truspec-Micro CHNS 630-200-200. Fourier transform infrared (FTIR) spectra of the materials were collected using a Bruker Optics Tensor 27 spectrometer (Bruker, Billerica, MA, USA) coupled to a horizontal attenuated total reflectance (ATR) cell, using 256 scans at a resolution of 4 cm^−1^. The nanomaterials were analyzed by powder X-ray diffraction (XRD) using a Rigaku Geigerflex Dmax-C diffractometer (Rigaku, Tokyo, Japan) equipped with a CuKα monochromatic radiation source with a step size of 0.026° and time per step of 350 s. The morphology and size of the particles were analyzed by electron microscopy using a Hitachi HD-2700 scanning transmission electron microscope (Hitachi, Tokyo, Japan) operating at 200 kV. Samples for electron microscopy analysis were prepared by evaporating the diluted suspensions of the particles on a copper grid coated with an amorphous carbon film. The surface charge of the nanoparticles was assessed by zeta potential measurements through electrophoretic light scattering performed using a Zetasizer Nano ZS instrument equipped with a HeNe laser operating at 633 nm and a scattering detector at 173 degrees, from Malvern Instruments (Malvern, UK). The measurements were performed in aqueous suspensions of the particles using a disposable folded capillary cell. The magnetic measurements were performed using the Quantum Design SQUID MPMS3 (Quantum Design, San Diego, CA, USA) both in VSM and DC modes, as a function of the applied magnetic field (from +50 to −50 kOe), at 300 K and 5 K. The magnetic dc susceptibility was recorded at increasing temperatures (from 10 to 300 K) with an applied H_app_ = 100 Oe after initial cooling in the absence of the field (ZFC procedure) and in the presence of H_app_. To estimate the Fe_3_O_4_ content of the coated particles, 10 mg of particles were digested in 20 mL of hydrochloride acid (37%). Afterwards, the solutions were analyzed using atomic absorption spectrophotometry (AAS) in a Perkin Elmer Analyst 100 apparatus (PerkinElmer, Waltham, MA, USA) to assess the Fe content.

### 3.4. Doxorubicin Loading and Release Studies

Doxorubicin hydrochloride solutions of known concentration were prepared in sodium phosphate buffer (0.2 M, pH 6). Nanoparticles were accurately weighed and incubated in 2 mL of the solution (1.25 mg NPs/mL buffer) and the mixtures were vertically stirred (Heidolph Reax 2, Heidolph Instruments, Schwabach, Germany, 30 rpm) for 24 h at room temperature in dark. The nanoparticles were separated magnetically and the solution’s remaining doxorubicin content ([DOX]_final_) was determined via UV-Vis spectrophotometry at 480 nm (Cintra 303 GBC Scientific Equipment, Melbourne, Australia). The loading efficiency and the nanoparticle capacity were calculated using equations 1 and 2, respectively [17]. Control experiments using loading solutions without nanoparticles were also carried out in parallel to exclude adsorption or degradation phenomena as the cause for the decrease of DOX concentration in the solutions. A quantification curve for doxorubicin was drawn from several standards—5, 10, 20, 30, 40 and 50 μg/mL, on the mentioned buffer (Figure S1—Supporting Information).

(1) $$Loading\;Efficiency\;\left(\%\right)=\frac{{\left[DOX\right]}_{initial}-{\left[DOX\right]}_{final}}{{\left[DOX\right]}_{initial}}\;\times \;100$$

(2) $$Nanoparticle\;Capacity\;\left(m_{DOX}/m_{NP}\right)=\frac{m_{loaded\;doxorubicin}}{m_{nanoparticles}}$$

To obtain the doxorubicin release profiles, the loaded nanoparticles (2.5 mg NP) were transferred to 10 mL of sodium phosphate buffer (0.2 M) with pH value of 4.2, 5.0 and 7.4, representing the pH of intracellular acidic endosomes, the pH of the tumor microenvironment, and the pH of blood, respectively. The dispersion was vertically stirred (Heidolph Reax 2; 30 rpm) at 37 °C for 48 h [17]. At specific time intervals an aliquot (1 mL) of the solution was taken and replaced by equal volume of fresh buffer. The nanoparticles were magnetically separated to the bottom before taking the supernatant sample. The sample was then analyzed using UV-Vis spectrophotometry (480 nm) to assess DOX concentration.

### 3.5. Cell Studies

#### 3.5.1. Cell Culture and Treatment

The breast cell lines used were: MCF7 (an estrogen receptor (ER)-positive human breast epithelial adenocarcinoma cell line; ATCC HTB-22; passage numbers 79–82), MDA-MB-231 (a triple negative human breast adenocarcinoma cell line; ATCC HTB-26; passage numbers 47–50) and MCF12A (a non-tumorigenic epithelial cell line; ATCC CRL-10782; passage numbers 29–32).

Cells were maintained in a humidified atmosphere of 5% CO_2_–95% air and were grown in RPMI 1640 medium supplemented with 2 mM l-glutamine, 10 mM sodium bicarbonate, 15% heat-inactivated FBS and 1% antibiotic/antimycotic (MCF-7 and MDA-MB-231 cell lines) or DMEM:Ham’s F12 medium (1:1) supplemented with 20 ng/mL human epidermal growth factor, 100 ng/mL cholera toxin, 0.01 mg/mL bovine insulin, 500 ng/mL hydrocortisone, 5% heat-inactivated horse serum and 1% antibiotic/antimycotic (MCF12A cell line). For the assays, cells were seeded in 24-well culture dishes and used at 90% confluence. Cells were exposed to the treatment (or to the solvent—water) for 24 h in FBS-free culture medium.

#### 3.5.2. Cytotoxicity and Cell Proliferation Determination

Cytotoxicity and cell proliferation tests aimed at assessing the nanoparticles’ viability as a drug carrier towards cancer cells. Unloaded nanoparticles and doxorubicin were used in separate tests to determine the effectiveness of the loaded carrier compared to just the carrier itself and the free drug. MCF-7 and MDA-MB-231 human breast cancer cells and MCF12A human breast cells were exposed for 24 h to doxorubicin (free, or carrier loaded) at concentrations between 1 and 50 μM, and equivalent concentrations of unloaded nanoparticles.

Cell proliferation was determined through thymidine incorporation assay. Briefly, cells were incubated with ^3^H-thymidine (^3^HT) during the last 5 h of their 24 h exposure to the drug or nanoparticles, where ^3^HT is incorporated in new strands of DNA during cell division. Excess ^3^HT was removed with 300 mL of 10% TCA for 1 h at 4 °C, followed by drying for 30 min, and addition of 1 mol/L NaOH (280 μM /well), and the lysate was neutralized with 5 mol/L HCl prior to the addition of scintillation fluid. The radioactivity of the samples was then quantified by liquid scintillometry and expressed as incorporation of ^3^HT in mCi/mg protein (compared to a control).

Cellular viability was determined through l-lactic dehydrogenase (LDH) activity assay, expressed as the percentage of extracellular activity in relation to total cellular LDH activity [83]. After a 24 h exposure of the cells to drug or nanoparticles, cellular leakage of LDH into the extracellular culture medium was determined spectrophotometrically by measuring the decrease in absorbance of NADH (340 nm) during the reduction of pyruvate to lactate. To determine total cellular LDH activity, control cells were exposed to 0.1% (*v*/*v*) Triton X-100 for 30 min at 37 °C.

## 4. Conclusions

In conclusion, we reported here a promising drug delivery system comprising DOX loaded nanocarriers for anti-cancer therapy. These nanocarriers (Fe_3_O_4_@SiĸCRG) are composed of siliceous and carrageenan hybrid shells that coat superparamagnetic magnetite nanoparticles. It was found that the Fe_3_O_4_@SiĸCRG nanocarriers have a loading capacity for DOX between 3.3 and 12.3 wt%, which is comparable to other reported magnetic hybrid systems, and an optimal drug release profile in aqueous solutions—80% (tumor microenvironment pH) versus 25% (blood pH) in over 6 h. The underlying mechanism for the pH-dependent DOX release behavior relies on a cation exchange process that takes into account the presence of ammonium groups in the drug and the sulfonated moieties of ĸ-carrageenan. Therefore, by controlling the surface chemistry of the Fe_3_O_4_@SiĸCRG nanocarriers, it provides a way to adjust the DOX release upon exposure of the nanocarriers to biological environments of distinct pH. Since this is of acute relevance for DOX based cancer therapies, the drug loaded nanocarriers were also tested in cellular cultures (cancer and non-cancer cell lines) and showed clear cytotoxic and antiproliferative activity. Future work should include clinical tests to confirm the enhanced permeability and retention effect and optimal pH-responsiveness apply in physiological conditions. Finally, the superparamagnetic quality of the Fe_3_O_4_@SiĸCRG nanocarriers opens the way towards the development of theranostic agents, namely by assessing their MRI-responsiveness.

## Figures and Tables

**Table 1:** Elemental analysis and textural properties of citrate-capped Fe_3_O_4_ and Fe_3_O_4_@SiκCRG nanoparticles (D—core diameter, T—coating thickness, S_BET_—specific surface area, C,H,S—carbon, hydrogen and sulphur content).

Sample	*D* (nm)	*T* (nm)	C (%)	H (%)	S (%)	S_BET_ (m^2^·g^−1^)
**Fe_3_O_4_**	10 ± 2.5		6.55	1.28		152.00
**Fe_3_O_4_@SiκCRG**	10 ± 2.5	5.2 ± 2.1	13.85	2.27	1.27	13.94

**Table 2:** Magnetic parameters for Fe_3_O_4_ and Fe_3_O_4_@SiκCRG particles, at 300 K.

Material	*Ms* (emu/g_sample_)	*Ms* (emu/g_Fe3O4_)	*Mr* (emu/g_Fe3O4_)	*Hc* (Oe)
**Fe_3_O_4_**	23	25	1.2	31
**Fe_3_O_4_@SiĸCRG**	15	27	1.9	32

## Supplementary Materials

The following are available online, Figure S1: Calibration curve to determine the DOX concentration using UV-Vis spectroscopy at 480 nm, Table S1: Doxorubicin loading efficiency and nanoparticle capacity at variable DOX concentration (pH = 6, C_NP_ = 1.25 mg/mL), Figure S2: Field Dependent Magnetization Curves (without normalization) of Fe_3_O_4_ nanoparticles (left) and Fe_3_O_4_@SiκCRG nanoparticles (right), Figure S3: Speciation of DOX, Figure S4: Doxorubicin release profiles over 48 h, with corresponding fitting using the Weibull model, Table S2: Parameters α and β, as estimated from the application of the Weibull model to the DOX release data, and coefficient of determination (R^2^).
