# Supplementary material for: Magnetic Driven Nanocarriers for pH-Responsive Doxorubicin Release in Cancer Therapy
Source: Molecules. 2020 Jan 14;25(2):333. doi: 10.3390/molecules25020333 (PMC7024164; doi:10.3390/molecules25020333)
Supplement: Supplementary file 1 [file molecules-25-00333-s001.pdf]

molecules

MDPI

Supporting Information

Magnetic Driven Nanocarriers for pH-Responsive Doxorubicin Release in Cancer Therapy

João Nogueira [1], Sofia F. Soares [1], Carlos O. Amorim [2], João S. Amaral [2], Cláudia Silva [3,4], Fátima Martel [3,4], Tito Trindade [1] and Ana L. Daniel-da-Silva [1,*]

1 CICECO-Aveiro Institute of Materials, Department of Chemistry, University of Aveiro, 3810-193 Aveiro, Portugal; jh.nogueira@ua.pt (J.N.); sofiafsoares@ua.pt (S.S.); tito@ua.pt (T.T.)
2 CICECO-Aveiro Institute of Materials, Department of Physics, University of Aveiro, Aveiro, 3810-193, Portugal; amorim5@ua.pt (C.A); jamaral@ua.pt (J. A.)
3 Unit of Biochemistry, Department of Biomedicine, Faculty of Medicine, University of Porto, Porto, 4200-319, Portugal ; rakelclaudia@hotmail.com (C.S.); fmartel@med.up.pt (F.M.)
4 i3S-Instituto de Investigação e Inovação em Saúde, Universidade do Porto, Porto, 4200-135, Portugal
* Correspondence: ana.luisa@ua.pt; Tel.: +351-234-370-368

Received: 11 December 2019; Accepted: 10 January 2020; Published: 14 January 2020

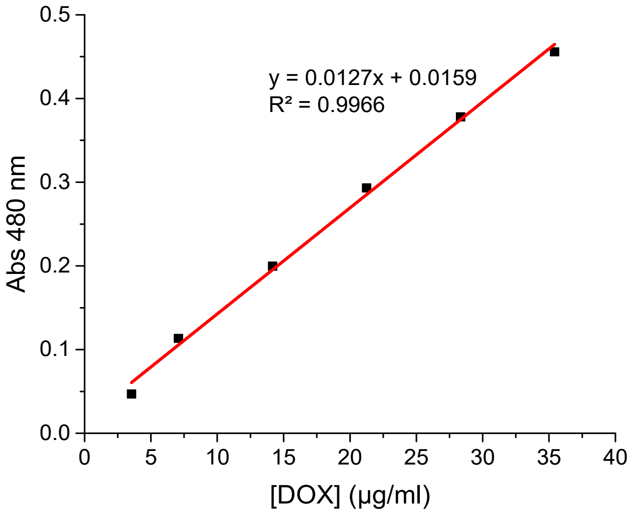

Figure S1. Calibration curve to determine the DOX concentration using UV-Vis spectroscopy at 480 nm.

Table S1. Doxorubicin loading efficiency and nanoparticle capacity at variable DOX concentration (pH = 6, C_{NP} = 1.25 mg/mL).

**$[DOX]_{initial}$ (μg/mL)**	**Efficiency (%)**	**Capacity (μg DOX/mg NP)**
110	37.25 ± 2.23	32.92 ± 1.97
120	50.37 ± 1.75	48.61 ± 1.69
190	55.04 ± 2.89	82.40 ± 4.33
350	43.77 ± 4.30	122.54 ± 12.03

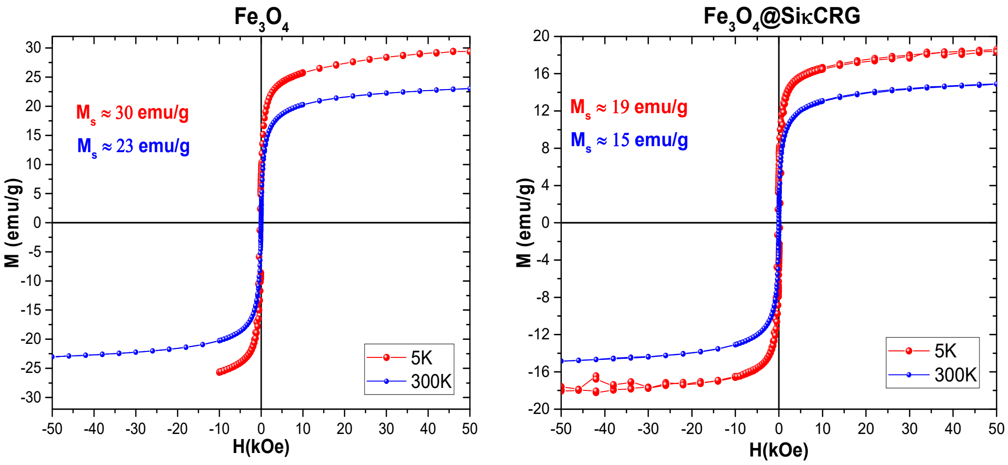

Figure S2. Field Dependent Magnetization Curves (without normalization) of Fe_3O_4 nanoparticles (left) and Fe_3O_4@SiκCRG nanoparticles (right).

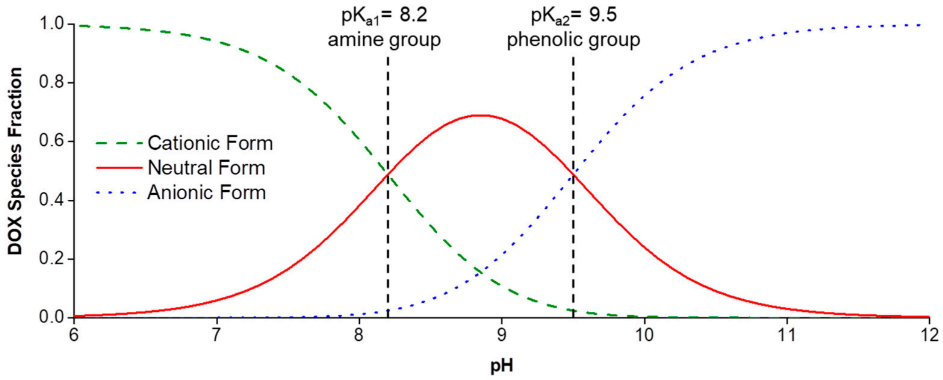

Figure S3. Speciation of DOX.

__Release Kinetics Modeling__

To analyse the release kinetics, the Weibull model [S1, S2] was fitted to the experimental data. The Weitbull model is described by equation (S1)

$$m = 1 - e^{\left(-\frac{(t-T_i)^{\beta}}{\alpha}\right)} \qquad \text{(equation S1)}$$

where *m* is the cummulative fraction of released drug (0 to 1), t is the release time, *α* is the time process,Ti is the lag time, in most cases zero, and β, the shape parameter, characterizes the curve as exponential (b = 1), S-shaped with upward curve followed by turning point (b > 1), or parabolic with higher initial slope, after that consistent with the exponential (b < 1).

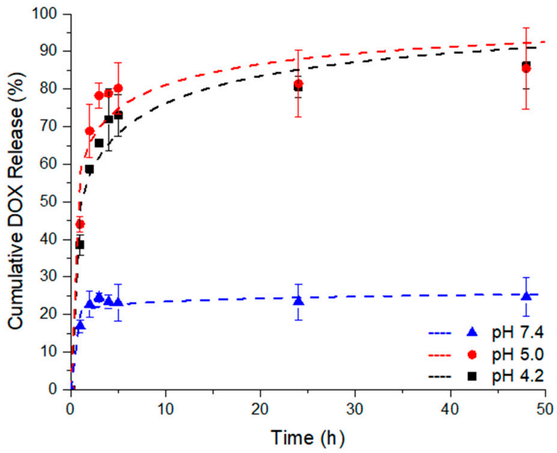

Figure S4. Doxorubicin release profiles over 48 hours, with corresponding fitting using the Weibull model.

Table S2. Parameters α and β, as estimated from the application of the Weibull model to the DOX release data, and coefficient of determination (R^2).

Parameter	pH 4.2	pH 5.0	pH 7.4
α	1.473	1.127	4.296
β	0.324	0.272	0.058
R^2	0.9576	0.9262	0.9468

References

1. Langenbucher, F. Linearization of dissolution curves by the Weibull distribution. *J. Pharm. Pharmacol.* **1972**, *24*, 979–981.

2. Costa, P.; Sousa Lobo, M.J. Modeling and comparison of dissolution profile. *Eur. J. Pharm. Sci.* **2001**, *13*, 123–133.

© 2020 by the authors. Licensee MDPI, Basel, Switzerland. This article is an open access article distributed under the terms and conditions of the Creative Commons Attribution (CC BY) license (http://creativecommons.org/licenses/by/4.0/).
